# How digital health technologies promote healthy life in the Post-COVID-19 Era: evidences from national survey on Chinese adolescents and youngsters

**DOI:** 10.3389/fpubh.2023.1135313

**Published:** 2023-05-09

**Authors:** Xiaojing Li, Min Zhang

**Affiliations:** School of Media & Communication, Shanghai Jiao Tong University, Shanghai, China

**Keywords:** Post-COVID-19 Era, digital health technologies (DHTs), behavioral regulation, healthy lifestyle, Chinese adolescents and youngsters

## Abstract

The rapid development of intelligent technologies coupled with the stay-at-home trends in the Post-COVID-19 Era has significantly changed youth's health behavior as well as reshaped their lifestyles. Digital health technologies (DHTs) have been more and more used for health management among youngsters. However, little was known about the use of DHTs among youths and its consequences on their health, especially in developing countries like China. Inspired by behavior intervention technology (BIT) model, this study examined the underlying mechanisms of use and social interactions of DHTs on Chinese adolescents' and youngsters' healthy lifestyles and mental health, using a representatively national survey among high school and freshman students in China (*N* = 2,297). It found that use of DHTs had a significantly positive effect on Chinese youths' healthy lifestyles and mental health, with behavioral regulation as a mediator. However, social interactions of DHTs were negatively associated with their mental health. The findings contribute to a better guidance on health promotion, as well as the enhanced design of DHTs' products.

## 1. Introduction

At the end of 2019, a sudden epidemic swept the world. During the COVID-19 pandemic, the imposition of lockdown measures was unprecedented and dramatically changed the common life of the public (1). People have significantly less access to outdoor exercise or professional fitness equipment due to outdoor movement restrictions (2). COVID-19 pandemic has also created barriers to physical activities among adolescents and youngsters, whose health management has entered a dilemma (3). Several studies have investigated the adverse mental health consequences associated with COVID-19 pandemic (4), and concluded that the pandemic negatively affected people's mental health (e.g., increase in post-traumatic stress symptoms, depression, anxiety, and insomnia) by influencing multiple factors (5, 6). Among them, a decrease in physical activities is an important reason (7, 8). It showed that the lifestyle changes caused by COVID-19 pandemic had a deep impact on people's physical and mental health.

Meanwhile, there has been increasingly academic interests in digital health technologies (DHTs, such as Fitbit, Keep, Smartwatch, etc.) as tools for health management in recent years (9). Especially during COVID-19 pandemic, the use of DHTs for exercise and physical activities became more and more popular in public healthy life (10). DHTs received extensive attention in the fields of clinical medicine, communication, sociology, and computer science. Academic topics existed in clinical assessment and intervention for mental illness (11) and a variety of chronic illnesses (12), conducting health promotion activities, monitoring public health (13), and so on.

Although there were several studies which have explored the relationship between the use of DHTs and users' health behaviors or healthy lifestyles (14, 15), the effectiveness of DHTs for health behavior was controversial (16). For instance, one meta-analysis found that smartphone apps had a non-significant and positive influence on participants' physical activity (17), while another meta-analysis suggested that only about 20% of studies found that the app had a significant effect on users' health behavior change, and nearly 45% of the studies showed the opposite conclusions (18). Also, there was a lack of study considering the underlying mechanism between the use of DHTs and healthy lifestyles.

In addition, most of the prior findings aimed at adults and used convenience samples of adults (19), while there was still little concerns on adolescents' and youngsters' DHTs usage in their health management (20). Given that engagement with new technology is highly valuable for adolescents' lives (21), and adolescents are increasingly using DHTs for exercise logging, diet management, sleep monitoring, social interactions, and health behavior (22), it's critical to examine how the use and interactions of DHTs may affect adolescents' and youngsters' health behavior and improve their mental health.

Therefore, the aim of this study was to explore the impact and underlying mechanisms of the use and social interactions of DHTs on adolescents' and youngsters' healthy lifestyles and mental health, based on a national sample consisting of Chinese students.

## 2. Literature review

### 2.1. Overall use of DHTs among adolescents and youngsters

In the age of digital technologies, more and more adolescents are using digital technologies to explore health topics and conduct health management (23). National surveys showed that fully 95% of adolescents had access to a smartphone (24). More than 80% of 1,300 adolescents reported having searched for health information online, and nearly two-thirds (64%) said they have used mobile apps related to health, including for fitness, nutrition, sleep and so on (25). Many adolescents received daily step counts from wristwatches (20), and nearly a quarter (23%) of 1,156 adolescents had downloaded apps related to exercise or fitness, in which 14% had downloaded nutritional apps (26).

Thousands of “healthcare and fitness” apps for mobile clients provided technical support for health management (27). Fitbit was the main brand of DHTs used in western countries, and Keep was the most widely used DHT among Chinese adolescents. These apps allowed users to set targets, enhance self-monitoring, and raise awareness, which seemed to be a promising tool for health promotion (28). A variety of DHTs have been used to monitor physical activities (29, 30), control weight (31, 32), and plan nutrition (33) among adolescents and youngsters.

DHTs usually consisted of mHealth, wearables, and digital devices for health management and promotion (34–36). Among them, mHealth comprised applications on mobile devices designed to promote health (10, 36, 37). Wearable devices were designed to be worn on the user's body, using sensors to track the wearer's movements or biometric data, which could provide feedback to motivate health behavior change (19, 38).

### 2.2. DHTs, healthy lifestyles, and mental health

A healthy lifestyle is effective in reducing the risk of serious illness or premature death (15). It has been proved to be predicted by healthy diet, healthy level of physical activities, healthy body weight, non-smoking, and moderate alcohol intake, etc. (39, 40). Many studies have focused on physical activities, nutrition, and stress management as important measures of a healthy lifestyle (41), in which symptoms of depression was an important predictor of mental health (42, 43). This study aimed to explore the associations among uses of DHTs, healthy lifestyle, and mental health on the basis of previous findings.

Prior studies showed that the use of digital interventions may have the potential to improve individuals' diet, physical activities, sleep, and weight control (9, 20, 30). DHTs also reduced one's stress or physical illness (44). For example, Finkelstein et al. (45) examined the efficacy of activity trackers (Fitbit) to promote physical activities through a randomized controlled trial. Yen (41) investigated the effectiveness of smart wearable devices for shaping a healthy lifestyle and improving wellbeing. Chung et al. (46) tested the effects of the mHealth app, Twitter, and fitness trackers on promoting a healthy lifestyle. It showed that the mHealth interventions were useful in increasing participants' steps and vegetable/fruit intake while reducing their sugar-sweetened beverage intake.

Furthermore, previous studies have shown that DHTs combined with social influence were effective in increasing users' levels of physical activities (47, 48). Sharing fitness situations or data with other users was an important aspect of social influences (49). In addition, a review of the relevant literature has revealed that social networks were one of the key features that promoted physical activities, including sharing experiences, information, and achievements among friends (50). Also, the use of DHTs including social components could promote positive health behaviors (36).

**Figure 1:**
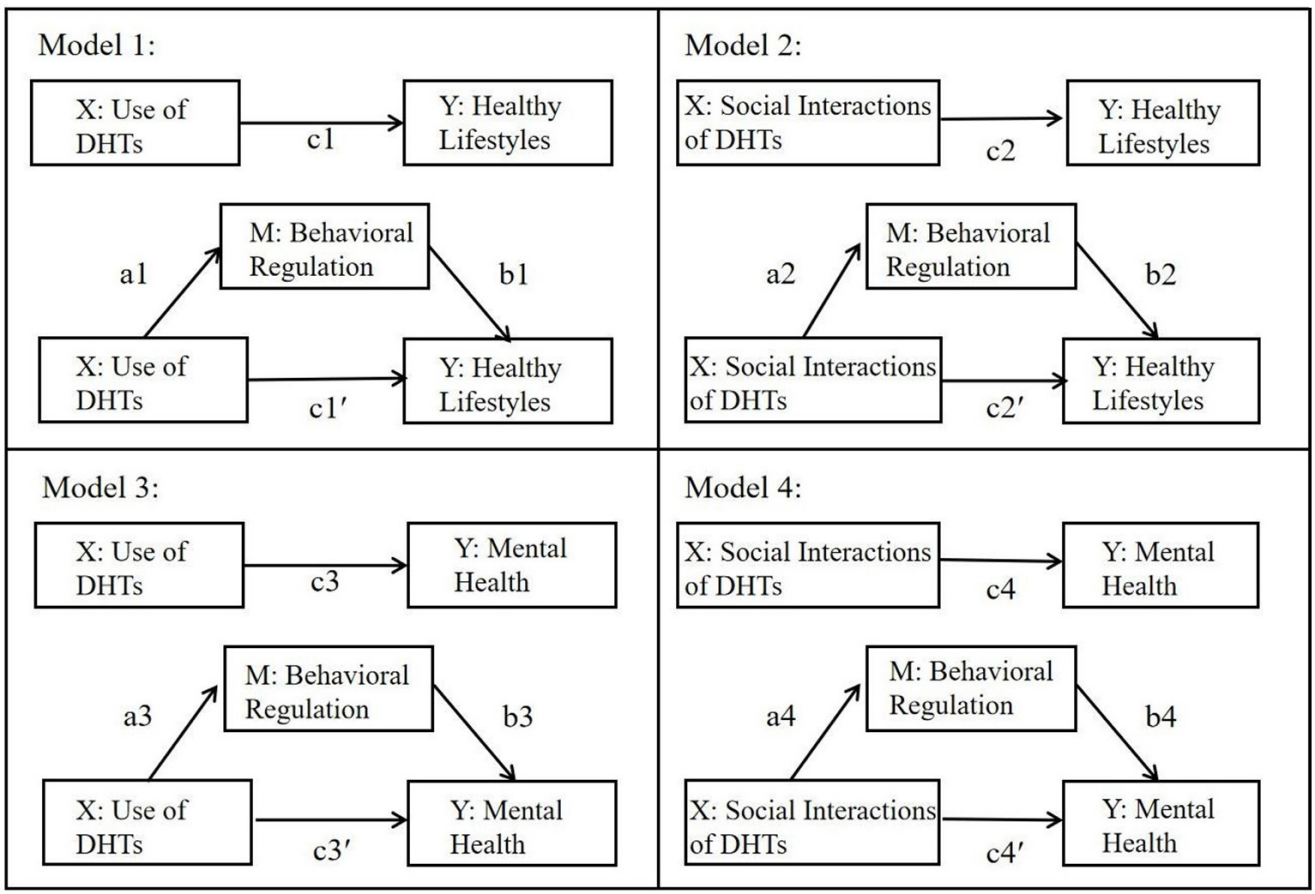
The research framework and conceptual models of the performed mediation analyses. X = independent variables, M = mediating variables, and Y = dependent variables. Coefficient c designates the total effect of the respective X on Y (ignoring M); coefficient a is the effect of X on M, coefficient b is the effect of M on Y (controlling for X); coefficient c′ designates the direct effect of X on Y when M is controlled; a × b reflects the indirect or mediation effect.

Therefore, a set of following hypotheses were articulated (as Figure 1 shows):

H1a: Use of DHTs is positively correlated with healthy lifestyles.
H2a: Social interactions of DHTs are positively correlated with healthy lifestyles.
H3a: Use of DHTs is positively correlated with mental health.
H4a: Social interactions of DHTs are positively correlated with mental health.

### 2.3. BIT model and behavior change interventions

With the rapid development of science and technology, technological innovations continued to reshape people's cognition and life. The behavior intervention technology (BIT) model explained changes in healthy behaviors caused by technical interventions. BIT referred to behavioral and psychological interventions through devices (e.g., computers, mobile phones, tablets, and sensors) and software (such as mobile health apps and Internet sites) (51), aiming at changing behaviors and cognitions related to physical health, mental health, and wellness (52). The overall BIT model consisted of four parts, say, aims, elements, characteristics, and workflow (53).

It revealed that one's self-regulation played an important role in the process of technological products triggering changes in healthy behaviors. Self-awareness, self-management, and self-efficacy were also important self-binding strategies of BIT for successful interventions to promote healthy behavioral changes (41, 54). Studies have shown that the degree to which behavior change goals are internalized and integrated could significantly affect the results of behavioral change (55).

Behavioral regulation included introjected regulation and identified regulation. Introjected regulation implied that the behavior was done for controlling reasons (to avoid guilt or to please others), whereas identified regulation implied that one has accepted the behavior as important and meaningful, i.e., self-determined (56). Both of them might lead to behavioral changes. Thus, this study took behavioral regulation as an important mediator.

These four hypotheses were put forward accordingly:

H1b: Behavioral regulation mediates the relationship between use of DHTs and healthy lifestyles.
H2b: Behavioral regulation mediates the relationship between social interactions of DHTs and healthy lifestyles.
H3b: Behavioral regulation mediates the relationship between use of DHTs and mental health.
H4b: Behavioral regulation mediates the relationship between social interactions of DHTs and mental health.

As mentioned above, inspired by BIT model, we examined the underlying mechanisms of use of DHTs on adolescents' healthy lifestyles and mental health. To fill the gaps in existing studies, we incorporated the use and social interactions of DHTs as predictors and behavioral regulation as a mediator in this work. Figure 1 showed the whole research framework of the performed mediation analyses, based on a nationally representative sample of Chinese students.

## 3. Methods

### 3.1. Participants

To test the hypotheses, a nationwide questionnaire survey was conducted in China from May 2021 to June 2021, using a cluster-randomized sampling method to obtain representative national samples. The subjects of the survey were adolescents and youngsters aged from 15 to 24 years old (*M* = 18.71, *SD* = 1.814). Sixty-six trained investigators participated in the questionnaire survey. Finally, we received 3,330 responses from 31 provinces and metropolitans across the whole China. After removing respondents that have not used any DHTs or missed more than 10% of the items, a total of 2,297 responses were obtained, with effective rate of 68.98%.

### 3.2. Procedures

Before the survey, we validated the applicability and reliability of the measurement scales through a comprehensive literature review. Ten adolescents and youngsters (both boys and girls) from high schools and colleges were invited for a pretest. After they completed the questionnaire, an interview was conducted to collect their opinions and suggestions. Based on their feedback, some problematic items were modified accordingly to improve the accuracy of the questionnaire, and then the scales were finalized for this study. One high school and one university were selected as the target schools in each of the 31 provinces or metropolitans across China, via a cluster-randomized sampling method. Students at the target schools were interviewed anonymously in their own classrooms. All the participants completed the survey after the trained interviewers provided detailed instructions to them and all the informed consents were obtained from the schools, teachers, and students. Investigational sessions lasted approximately 20 min, in which cross-sectional data were collected.

### 3.3. Measurement

#### 3.3.1. Demographics

The gender, age, and grade of the students were included in this survey. Besides, all the participants were asked to calculate and report their own BMI, which was measured by their height and weight.

#### 3.3.2. Use of DHTs

To measure the extent of participants' the use of DHTs, they were asked two questions: “For the above-mentioned devices/APPs that you used most frequently, approximately how many times did you use them per week in the past month?” and “How many minutes did you use each time?” Participants filled in the real numbers based on their usage, and the data were analyzed by dividing the answers into six levels (1 to 6) in equal intervals. The final score of DHTs use was calculated based on the mean of two items.

#### 3.3.3. Social interactions of DHTs

Participants' social interactions of DHTs were measured by asking: “In the past month, how often did you share your sports-related situations (including and not limited to text, screenshots, etc.) via the devices/apps on social media platforms (such as WeChat Moments, Weibo, QQ zone, etc.)?”, and “In the past month, how often did you talk to others about your use of the devices/apps and other related topics?”, with the answers ranging from 1 = never, 2 = once, 3 = 2–3 times, 4 = at least once per week, to 5 = two or more times per week. The two items were averaged to form the score of social interactions of DHTs. A higher score represented higher level of social interactions.

#### 3.3.4. Behavioral regulation

Behavioral regulation was measured by 12 items derived from the Behavioral Regulation in Exercise Questionnaire (BREQ) (57), which included “I value the benefits of exercise”, “I feel guilty when I don't exercise” etc., with the choices 1 = not true for me, 2 = not very true for me, 3 = neither true nor false for me, 4 = mostly true for me, to 5 = very true for me. A higher score indicated higher level of behavioral regulation. Finally, the 12 items were averaged to form the behavioral regulation scale (*M* = 2.70, *SD* = 0.76, α = 0.88).

#### 3.3.5. Healthy lifestyles

This construct was measured by 23 items, which were modified based on the health Promotion Lifestyle Profile Scale (HPLP-S) (58), consisting of the items concerning respondents' nutrition, physical activities, stress management, etc., with the answer choices 1 = not true for me, 2 = not very true for me, 3 = neither true nor false for me, 4 = mostly true for me, to 5 = very true for me. Average scores of 23 items were calculated to indicate the level of respondents' healthy lifestyles (*M* = 3.02, *SD* = 0.66, α = 0.88).

#### 3.3.6. Mental health

Mental health was measured by the 10-item Chinese version of Center for Epidemiologic Studies Depression Scale (CESD-10) (59, 60), which has been widely used to measure depressive symptoms in the general population. It consisted of items such as “I was bothered by things that usually don't bother me”, “I could not get going”, and so on, with the choices 1 = Rarely or none of the time, 2 = Some or a little of the time, 3 = Occasionally or a moderate amount of time, to 4 = Most or all of the time. Each item weighted equally and all items were added up to form the mental health index (*M* = 10.21, *SD* = 5.60, α = 0.81). Higher scores represented more severe depressive symptoms.

### 3.4. Statistical analysis

Descriptive statistics were used to assess respondents' demographic characteristics (including gender, age, grade, and BMI) and the overall usage of DHTs. Pearson correlations (two tails) were used to examine the correlations between independent variables and dependent variables. In order to test the mediation effects of behavioral regulation, four mediation models were employed. Descriptive statistics and Pearson correlations were calculated with IBM SPSS 25; mediation effect analyses were tested using the PROCESS version 3.5 (Model 4) proposed by Hayes (61); standardized, unstandardized, and 95% confidence intervals for the path coefficients were estimated using 5,000 bootstraps; the significance level used for all statistical tests was 0.05.

## 4. Results

### 4.1. Descriptive statistics

Tables 1, 2 displayed descriptive statistics and correlations among the variables. Total of 2,297 valid respondents included 1,179 males (51.3%) and 1,118 females (48.7%). The average age was 18.71 years (*SD* = 1.814). The majority of respondents' BMI was in the healthy category (62.1%). As regards to the use of DHTs, “KEEP” had the highest usage rate (75.9%), followed by smartwatches (39.1%), Sports World Campus (13.3%), etc. In addition, exercise record (time, steps, distance, etc.) (62.8%) and exercise guidance (46.6%) were the most frequently used functions.

**Table 1:** Descriptive statistics (*N* = 2,297).

	***n***	**%**
**Gender**		
Male	1179	51.3
Female	1118	48.7
Age (*M* = 18.71, *SD* = 1.814)		
15–17	687	29.9
18–20	1,227	53.4
21–24	383	16.7
**BMI**		
Underweight	477	20.8
Healthy	1,426	62.1
Overweight	284	12.4
Obesity	67	2.9
**Types of digital health technologies used**		
Keep	1,744	75.9
Smartwatch	899	39.1
Sports World Campus	305	13.3
Boohee	240	10.4
Codoon	174	7.6
FitTime	98	4.3
Other apps not listed	248	10.8
**Main functions used**		
Exercise record (time, steps, distance, etc.)	1,442	62.8
Exercise guidance	1,070	46.6
Body index monitoring (heart rate, etc.)	880	38.3
Punch card check-in	868	37.8
Sleep monitoring	762	33.2
Diet management	582	25.3
Other functions not listed	77	3.4

BMI, body mass index.

**Table 2:** Correlations of all variables.

	**1**	**2**	**3**	**4**	**5**
1. Use of DHTs.	-				
2. Social interactions of DHTs.	0.344^***^	-			
3. Behavioral regulation.	0.159^***^	0.202^***^	-		
4. Healthy lifestyles.	0.160^***^	0.106^***^	0.494^***^	-	
5. Mental health.	−0.016	0.105^***^	0.096^***^	−0.18^***^	-

^***^p<*0*.001.

### 4.2. The use and social interactions of DHTs, behavioral regulation, and healthy lifestyles

As for model 1 and model 2, the results showed that both use of DHTs (β = 0.188, *t* = 9.333, *p*<*0*.001) and social interactions of DHTs (β = 0.146, *t* = 7.147, *p*<*0*.001) positively predict healthy lifestyles, which confirmed Hypothesis 1a and 2a. Analyzing the indirect effects, results revealed that behavioral regulation mediated the relationship between use of DHTs and healthy lifestyles (ab = 0.022, 95% *CI* = [0.164, 0.028]), also the relationship between social interactions of DHTs and healthy lifestyles (ab = 0.079, 95% *CI* = [0.062, 0.096]). Hence, Hypotheses 1b and 2b were supported. Nevertheless, the results also suggested that even after accounting for the mediating role of behavioral regulation, both use of DHTs (β = 0.107, *t* = 6.089, *p*<*0*.001) and social interactions of DHTs (β = 0.041, *t* = 2.252, *p* = 0.024) still had a positive impact on healthy lifestyles. Behavioral regulation accounted for 42.69% of the total effect in model 1 and 72.01% in model 2. The detailed results were indicated in Table 3.

**Table 3:** Four mediating models and results.

	**Standardized beta**	**SE**	**t**	**Indirect effect (95% CI)**	**Total effect (95% CI)**
Model 1:				0.022 (0.164 to 0.028)	0.052^***^ (0.041 to 0.063)
c1′ (Use of DHTs → healthy lifestyles)	0.107^***^	0.005	6.089		
a1(Use of DHTs → behavioral regulation)	0.161^***^	0.006	7.976		
b1(Behavioral regulation → healthy lifestyles)	0.497^***^	0.016	27.579		
Model 2:				0.079 (0.062 to 0.096)	0.11^***^ (0.079 to 0.139)
c2′ (Social interactions of DHTs → healthy lifestyles)	0.041^*^	0.014	2.252		
a2(Social interactions of DHTs → behavioral regulation)	0.209^***^	0.017	10.352		
b2(Behavioral regulation → healthy lifestyles)	0.503^***^	0.0165	27.0497		
Model 3:				0.009 (0.006 to 0.012)	0.004 (−0.008 to 0.008)
c3′ (Use of DHTs → mental health)	−0.043^*^	0.039	−2.062		
a3(Use of DHTs → behavioral regulation)	0.161^***^	0.006	7.976		
b3(Behavioral regulation → mental health)	0.276^***^	0.014	13.111		
Model 4:				0.030 (0.022 to 0.039)	0.065^***^ (0.042 to 0.088)
c4′ (Social interactions of DHTs → mental health)	0.064^**^	0.012	3.038		
a4 (Social interactions of DHTs → behavioral regulation)	0.209^***^	0.017	10.352		
b4 (Behavioral regulation → mental health)	0.262^***^	0.014	12.244		

All analyzes controlled the age, gender, grade, and BMI. ^*^p < 0.05, ^**^p < 0.01, ^***^p < 0.001.

### 4.3. The use and social interactions of DHTs, behavioral regulation, and mental health

Regarding model 3 and model 4, the result showed that use of DHTs couldn't directly predict mental health (β = 0.002, *t* = 0.092, *p* = 0.927), thus failing to support Hypothesis 3a. But social interactions of DHTs had a positive impact on depression (β = 0.119, *t* = 5.610, *p*<*0*.001), which meant negative association with mental health. The result was contrary to hypothesis 4a. Analyzing the indirect effects, results revealed that behavioral regulation mediated the relationship between use of DHTs and mental health (ab = 0.009, 95% *CI* = [0.006, 0.012]), also the relationship between social interactions of DHTs and mental health (ab = 0.030, 95% *CI* = [0.022, 0.039]). Hence, Hypotheses 3b and 4b were supported. Nevertheless, the results also suggested that after accounting for the mediating role of behavioral regulation, use of DHTs had a negative impact on depression (β = −0.043, *t* = −2.062, *p* = 0.039), which meant positive association with mental health. Meanwhile, social interactions of DHTs still had a positive impact on depression (β = 0.064, *t* = 3.038, *p* = 0.002), which meant negative association with mental health. Behavioral regulation accounted for 22.75% of the total effect in model 3 and 46.24% in model 4. The detailed results were shown in Table 3.

## 5. Discussion

The COVID-19 pandemic has proposed unprecedented challenges for human life. In the post-COVID-19 Era, people's physical and mental health has been destabilized (62, 63). At the same time, the COVID-19 pandemic has enhanced people's reliance on DHTs and promoted the diffusion of DHTs (64). A study showed that more than half of interviewees tried to use DHTs during lockdown period (10). In this scenario, it is quite of worth and significance to explore how use of DHTs might promote people's health nowadays.

Previous studies revealed that the COVID-19 pandemic has negatively impacted people's physical activities (3), which were highly correlated with one's health, physical appearance, and psychological benefits. However, few works were shed light on the role of health technologies on individual's wellness. We know that self-regulation could significantly influence one's health behavior in this process (65). Therefore, this work examined that whether the use and social interactions of DHTs could lead to healthy lifestyles and mental health, mediated by behavioral regulation, which was almost confirmed in this study. Given the far-reaching impact of DHTs on adolescents and youngsters, this study investigated the impacts of health technologies on their health based on a nationally representative sample of Chinese students, which offered important theoretical and practical implications.

### 5.1. Theoretical implications

This study provided important theoretical contributions to the literature on DHTs usage, healthy lifestyles, and mental health. Firstly, this work broadened our knowledge of the use of DHTs among Chinese adolescents and youngsters. Despite of some studies investigating the use and impacts of DHTs among adolescents, the majority of them were conducted in developed countries, particularly in United States (20). Little was known about the youth's conditions in developing countries like China. Therefore, it is necessary to conduct a large-scale national survey in China to further validate existing findings. This study provided insights and rationale for understanding the use and implications of DHTs in different contexts.

Secondly, the study also enhanced our comprehension of DHTs' impacts on Chinese youth's healthy life. Prior studies have demonstrated the effectiveness of DHTs in promoting healthy lifestyles, including more physical activities, healthier diets, and more regular sleep (30, 36). However, few studies have explored the underlying mechanisms of the impacts. Less was known about social interactions, an essential function of DHTs, in previous studies. More importantly, youth's health included not only physical health but also mental health (66). Thus, these variables were all taken into account in our study. The findings suggested that both the use and social interactions of DHTs had significant impacts on Chinese youths' healthy lifestyles, with behavioral regulation playing an important mediating role in the relationships.

Somewhat surprisingly, this study has discovered that a higher frequency of social interactions on DHTs implied lower levels of mental health. That is, the more you interacted, the more depressed you were. One possible explanation lay in that the lonely and depressed had stronger desire to obtain social support, and social interaction was positively correlated with social support (67). Thus the lonely individuals might prefer to online social interactions (68). It has also been argued that social interactions included both positive and negative dimensions, in which negative interactions were associated with worse mental health (69). It meant further detailed measurement and studies in future.

Thirdly, the study demonstrated the indispensable role of behavioral regulation between use of DHTs and youths' health. It has found that there was no evidence of a statistically direct effect of use of DHTs on mental health. Nonetheless, after considering the mediating role of behavioral regulation, the results changed and the use of DHTs was predictive of better mental health. In other words, not the more you exercised the less depressed you were, while the more you exercised with behavioral regulation, the less depressed you were. Previous findings on whether use of DHTs predicted individual's health were controversial (30). In this study, the role of behavioral regulation might provide novel information and scholarly knowledge on the impacts of the use of DHTs.

### 5.2. Practical implications

Based on the BIT model, this study validated the essential role of behavioral regulation in health behavior change caused by DHTs, which should be taken into account in future health promotion policies and the design of DHTs. On one hand, both China and WHO suggested that schools or parents urge students to be more physically active or adopt healthier lifestyles at the behavioral level (70, 71). However, these appeals didn't guide youths to develop internal motivation to promote their behavioral regulation and healthy lifestyles. Thus, it's quite necessary to encourage adolescents and youngsters to foster awareness and self-control of healthy lifestyles.

On the other hand, prior studies showed that one of the biggest concerns regarding DHTs was whether individuals sustain their engagement with the technology over time (20). The lack of behavioral regulation motivation was one of the reasons why many people were unable to use DHTs consistently. Therefore, it's quite significant to improve the design of functions related to facilitating behavioral regulation, like the function of “Clock-in” and incentives for continuous clock-in, etc.

### 5.3. Limitations and future studies

Several limitations required considerations. First, we measured social interactions of DHTs with two items. Yet the definition of social interactions was far more complex (69). Therefore, we suggested that the measurement of social interactions could be divided into both positive and negative dimensions in future studies. Second, we didn't fully consider cultural factors and social backgrounds related to social interaction among Chinese adolescents and youngsters in our research design, which was indeed one of the limitations of our study. In future study, we will consider incorporating more social and cultural variables based on your suggestions to further enrich the model, revealing the special and complex social interaction factors in Chinese society, and exploring their underlying mechanisms. Thirdly, the use of self-reported measures might underestimate or overestimate the results. Future works could consider a randomized controlled trial to obtain long-term data via a panel study, to obtain generalized conclusions about the effectiveness and sustainability of DHTs in changing youths' health behaviors and maintaining healthy lifestyles.

## 6. Conclusion

In conclusion, based on the BIT model, this study provided important insights into one of the pathways in which the use and social interactions of DHTs impacted Chinese adolescents' and youngsters' healthy lifestyles and mental health. The findings contributed to theoretical and practical guidance on youths' health promotion and education, as well as the design of DHTs' products.

## Data availability statement

The raw data supporting the conclusions of this article will be made available by the authors, without undue reservation.

## Ethics statement

The studies involving human participants were reviewed and approved by the Institutional Review Board of School of Media & Communication at Shanghai Jiao Tong University (protocol code B2021003S, approved at 2021.03.29). Written informed consent to participate in this study was provided by the participants' legal guardian or themselves. Participation was completely voluntary, and the participants could choose to quit at any time for any reason during the process of answering the questionnaire. Written informed consent to participate in this study was provided by the participants' legal guardian/next of kin.

## Author contributions

XL contributed to conceptualization, methodology, investigation, resources, writing—original draft preparation, writing—review and editing, project administration, and funding acquisition. MZ contributed to formal analysis, investigation, data analysis, and writing—original draft preparation. All authors have made a substantial, direct, and intellectual contribution to the work, and agreed to the published version of the manuscript.
